# Septal rupture with right ventricular wall dissection after myocardial infarction

**DOI:** 10.1186/1476-7120-3-33

**Published:** 2005-10-20

**Authors:** Carlos J Soriano, José L Pérez-Boscá, Sergio Canovas, Francisco Ridocci, Pau Federico, Ildefonso Echanove, Rafael Paya

**Affiliations:** 1 Department of Cardiology. Consorcio Hospital General Universitario de Valencia. Valencia, Spain; 2 Department of Cardiac Surgery. Consorcio Hospital General Universitario de Valencia. Valencia, Spain

**Keywords:** Septal rupture, myocardial infarction, transthoracic echocardiography, transesophageal echocardiography

## Abstract

**Background:**

In patients with inferior myocardial infarction, septal rupture generally involves basal inferoposterior septum, and the communicating tract between left and right ventricle is often serpiginous with a variable degree of right ventricular wall extension. Right ventricular wall dissection following septal rupture related with previous myocardial infarction has been reported in a very few cases, in many of them this condition has been diagnosed in post-mortem studies. In a recent report long-term survival has been achieved after promptly echocardiographic diagnosis and surgical repair.

**Case Presentation:**

We present a case of a 59-year-old man who had a septal rupture with right ventricular wall dissection after inferior and right ventricular myocardial infarction. Transthoracic echocardiography, as first line examination, established the diagnosis, and prompt surgical repair allowed long-term survival in our patient.

**Conclusion:**

Outcomes after right ventricular intramyocardial dissection following septal rupture related to myocardial infarction has been reported to be dismal. Early recognition of this complication using transthoracic echocardiography at patient bedside, and prompt surgical repair are the main factors to achieve long-term survival in these patients.

## Background

The occurrence of ventricular septal rupture after acute myocardial infarction is an uncommon complication in the reperfusion era [1], however, this condition implies a high mortality rate, even after surgical repair [2]. In patients with inferior myocardial infarction, septal rupture generally involves basal inferoposterior septum, and the communicating tract between left and right ventricle is often serpiginous with a variable degree of right ventricular wall extension [3]. Right ventricular wall dissection following septal rupture related to previous myocardial infarction has been reported in a very few cases [4-6], in many of them this condition has been diagnosed in post-mortem studies [4]. In a recent report long-term survival has been achieved after promptly echocardiographic diagnosis and surgical repair [6].

## Clinical Case

**Figure 1:**
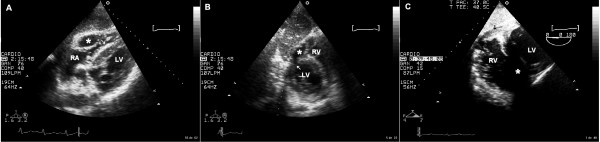
Panel A: transthoracic echocardiography, modified four chamber subscostal view showing the dissecting tract through right ventricular free wall forming an intramural neo-cavity (asterisk), the arrow indicates the re-entry point of dissecting tract into right ventricle chamber. Panel B: transthoracic echocardiography, modified short axis subcostal view showing rupture of the inferobasal septum at the point that originates the right ventricular wall dissection (arrow), asterisk indicates the dissected neo-cavity. Panel C: postoperative transesophageal echocardiography, transgastric short axis view showing thickened basal segments of the right ventricle and inferobasal septum without visible dissecting tract (black arrow), note increased echoreflectivity at this site. Acoustic shadow caused by prosthetic patch has been indicated (asterisk). Abbreviations: LV, left ventricle, RA, right atrium, RV, right ventricle.

**Figure 2:**
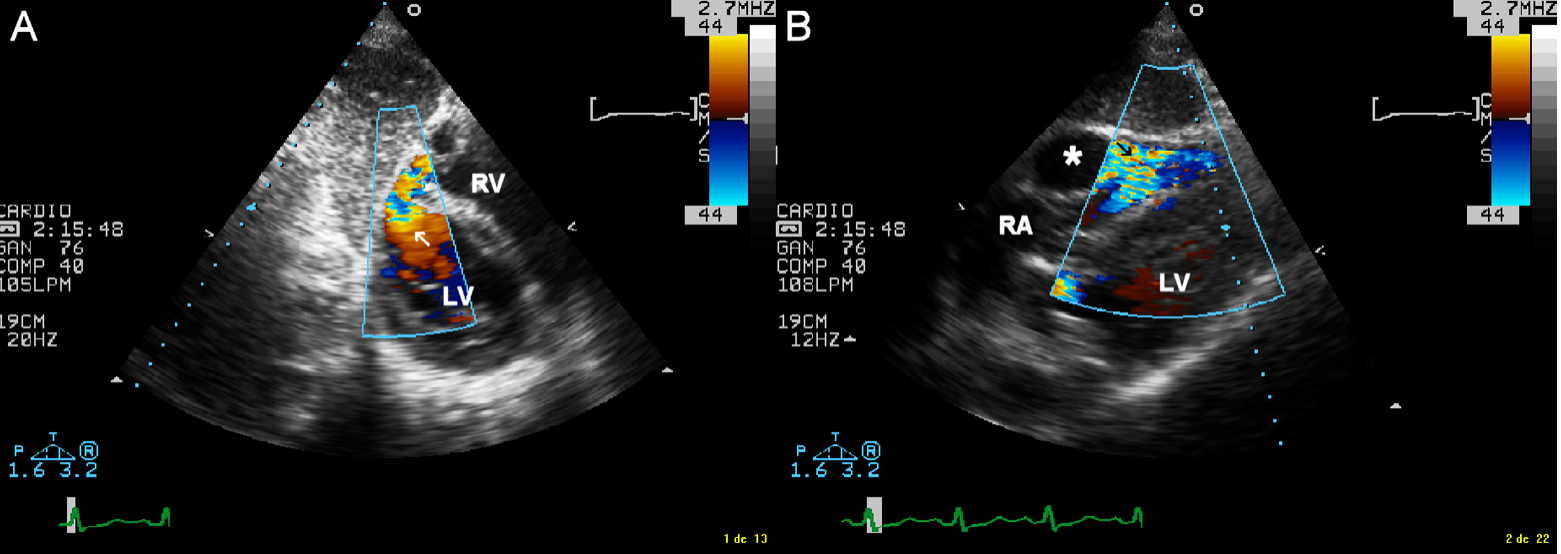
Panel A: transthoracic echocardiography, modified short axis subcostal view with color flow Doppler mapping showing left ventricular entry site of septal rupture (white arrow). Panel B: transthoracic echocardiography, modified four chamber subscostal view with color flow Doppler mapping showing septal rupture exit site in the right ventricular free wall (black arrow). Asterisk indicates the intramural neo-cavity in the right ventricular free wall. Abbreviations: LV, left ventricle, RA, right atrium, RV, right ventricle.

**Figure 3:**
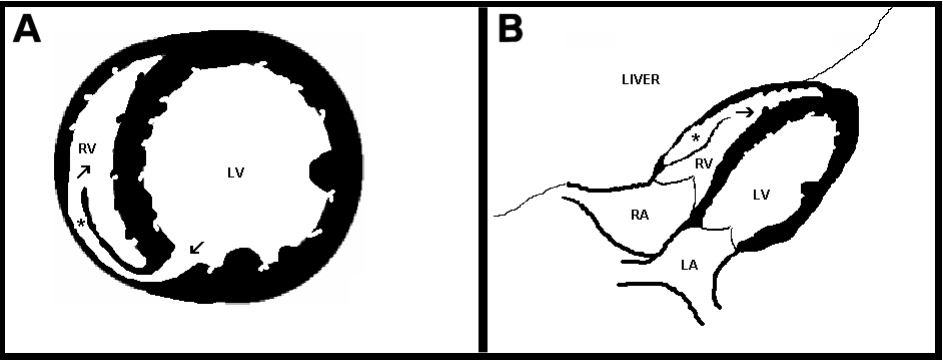
Schematic short axis (panel A) and four chamber subcostal (panel B) echocardiographic views showing the septal rupture location and dissecting tract trajectory through right ventricular wall. Blood flows from left ventricle (panel A, inferior arrow) into right ventricular wall creating an intramural neo-cavity (panel A and B, asterisk), and re-enters the right ventricle chamber (panel A, superior arrow, panel B arrow). Abbreviations: LV left ventricle, LA left atrium, RA right atrium, RV right ventricle.

A 59-year-old man was admitted to Coronary Care Unit because of suspected ST-segment-elevation myocardial infarction. The patient was complaining of typical coronary chest pain during the last twelve hours. He had a history of dyslipidemia, type 2 diabetes mellitus, smoking habit and a transient ischemic attack without any sensitive or motor squele one year ago. On admission, his blood pressure was 100/60 and heart rate was 110 beats per minute. Cardiac examination revealed jugular vein distension, and no significant heart murmurs. The ECG showed significant Q waves in II, III and aVf leads with mild ST-segment elevation in leads II, III, aVf and V4R. The chest radiograph revealed no cardiomegaly and clear lung fields. Transthoracic echocardiography was performed showing akinetic inferoseptal, inferior, and inferolateral segments with estimated left ventricular ejection fraction of 45%, right ventricle showed a global hipokinesia with severe systolic dysfunction and inferior vena cava plethora. The patient was initially treated with aspirin, low-molecular-weight heparin, dobutamine and saline infusions, and was scheduled for early catheterization. Coronary angiography showed total occlusion of right coronary artery proximal segment and two additional 70% stenoses in the first diagonal and obtuse marginal branches. Percutaneous revascularization was dismissed after a failed attempt of right coronary artery opening, then, the patient was treated using conservative medical therapy (aspirin 100 mg/day, clopidogrel 75 mg/day, simvastatin 20 mg/day and enalapril 10 mg/day), anticoagulation was maintained for 72 hours, and beta blocker therapy was not started because Mobitz I atrioventricular block phases were detected in continuous ECG monitoring. The later clinical outcome in the Coronary Care Unit was favourable, and the patient was discharged six days after to cardiology hospitalization unit. Nine days after hospital admission, the patient complained about sudden chest pain and rest dyspnea, his blood pressure was 80/40 and cardiac examination revealed a new harsh, holosystolic murmur along the left sternal border. Transthoracic echocardiography was immediately performed showing complex ventricular septal defect with a dissection tract that originated on left side of the basal inferoseptal akinetic segments, extended beyond the septum dissecting the right ventricular wall, and subsequently re-entered into the right ventricle chamber (figures 1, 2 and 3). No significant right ventricle outflow tract obstruction was present. The patient was scheduled for immediate surgical intervention, and hypothermic cardiopulmonary bypass with myocardial protection was established in the following two hours. The septal rupture was approached through the infarct, and prosthetic material (Gore-Tex^® ^patch) was used to reconstruct the septum, additionally, two bypasses using vein grafts were done in first diagonal and posterior descending arteries. Postoperative evolution was favourable with rapid resolution of cardiogenic shock situation. One month later transesophageal echocardiography was performed (figure 1, Panel C) showing neither right ventricle dissecting tract or residual shunt. Left ventricular ejection fraction was 55%, and right ventricular systolic function was only mildly depressed. At six-month follow-up the patient remains stable and without any cardiac symptoms.

## Conclusion

Outcomes after right ventricular intramyocardial dissection following septal rupture related to myocardial infarction has been reported to be dismal. As far as we know only two cases of this condition have been reported achieving long-term survival after surgical repair. According to Tighe et al [6], and taking into account that only two hours passed since symptoms' beginning to surgery, prompt echocardiographic diagnosis and surgical repair were probably the main factors that contributed to the long-term survival in our case. No additional surgical manoeuvre was performed on right ventricular dissected wall, however during postoperative evolution right ventricular intramyocardial neo-cavity resolved, as demonstrated by means of transesophageal echocardiography performed one month after surgical intervention (figure 1, panel C). Spontaneous apposition of right ventricular dissected layers and thrombosis of intramyocardial neo-cavity were simply facilitated after septal rupture closure.

When clinical suspicion of ventricular septal rupture complicating acute myocardial infarction is present, transthoracic and/or transesophageal echocardiography at patient bedside is the test of choice for early diagnosis and therapy guidance. Taking into account that complex forms of ventricular septal rupture with right ventricle involvement are critical prognostic factors [7], carefully echocardiographic recognition of complex dissecting tracts through the septum and right ventricular wall is of paramount importance. For this purpose, the use of unconventional echocardiographic views with color flow Doppler mapping, specially the use of subcostal views for visualize right ventricular free wall, allow to detect the left ventricular entry site and the right ventricular exit site of the septal rupture.

## Competing interests

All authors have read and approved submission of the manuscript, and no conflict of interest exists for any of the authors. The manuscript has not been published and is not being considered for publication elsewhere in whole or in part in any language

## Authors' contributions

CJS performed transthoracic echocardiography study, collected all the data and drafted the manuscript, JLP performed transesophageal echocardiography study and drafted the manuscript, SC performed surgical intervention, FR, PF, IE and RP, attended the patient and drafted the manuscript.
